# Degree of Impact of Tailor’s Bunion on Quality of Life: A Case–Control Study

**DOI:** 10.3390/ijerph18020736

**Published:** 2021-01-16

**Authors:** Victoria Mazoteras-Pardo, Ricardo Becerro-de-Bengoa-Vallejo, Marta Losa-Iglesias, Patricia Palomo-López, Daniel López-López, César Calvo-Lobo, Carlos Romero-Morales, Israel Casado-Hernández

**Affiliations:** 1 Grupo de Investigación ENDOCU, Departamento Enfermería, Fisioterapia y Terapia Ocupacional, Facultad de Fisioterapia y Enfermería de Toledo, Universidad de Castilla-La Mancha, 45071 Toledo, Spain; victoria.mazoteras@uclm.es; 2 Facultad de Enfermería, Fisioterapia y Podología, Universidad Complutense de Madrid, 28040 Madrid, Spain; ribebeva@ucm.es (R.B.-d.-B.-V.); cescalvo@ucm.es (C.C.-L.); israelcasado@yahoo.es (I.C.-H.); 3 Faculty of Health Sciences, Universidad Rey Juan Carlos, 28922 Alcorcón, Spain; marta.losa@urjc.es; 4 Department of Nursing, University Center of Plasencia, Universidad de Extremadura, 10600 Plasencia, Spain; 5 Research, Health and Podiatry Group, Department of Health Sciences, Faculty of Nursing and Podiatry, Universidade da Coruña, 15403 Ferrol, Spain; daniellopez@udc.es; 6 Faculty of Sport Sciences, Universidad Europea de Madrid, Villaviciosa de Odón, 28670 Madrid, Spain; carlos.romero@universidadeuropea.es

**Keywords:** foot, foot deformities, foot diseases, musculoskeletal diseases, quality of life

## Abstract

Tailor’s bunion (TB) disease should be considered one of the foot injuries that causes disability in feet as well as general health. This case–control descriptive study investigated and contrasted the effects of different TB types in a sociodemographic population using the Foot Health Status Questionnaire (FHSQ). A sample of 100 subjects with a mean age of 51.70 ± 17.78 years was recruited and requested to reply to a foot health survey. Results were self-reported. Subjects were scored. Participants with TB type III (TB3) registered lower scores for foot pain, foot function, footwear, and foot health. Physical activity and social capacity had higher scores, and vigor and general health were lower. A Kruskal–Wallis test was used for systematic differences between the FHSQ and different TB types. In all analyses, statistical significance was considered a *p*-value <0.05 with a 95% confidence interval. Statistically significant differences were found between all domains of the FHSQ and TB, except for the social capacity domain and vigor. The FHSQ is an important measurement tool in TB subjects, showing that factors such as sex, age, and footwear used throughout an individual’s life are significantly associated with the development of TB3 and its influence on foot pain and foot health.

## 1. Introduction

Bunionette, or tailor’s bunion (TB), is a foot disorder with a protrusion on the lateral broadside of the forefoot. This foot disorder is defined as a deformity that occurs in the axis between the fifth metatarsal and the little toe, thus producing a deviation of the fifth metatarsal head outwards and an inward deviation of the little toe [1]. A multifactorial etiology produces a plantar and dorsolateral prominence of the fifth metatarsal head [2]. The reference to the tailor’s occupation originated from their sitting while crossing their legs most of the day, with the outside of their feet brushing against the floor. This constant friction generated a painful bulge at the base of the little toe and high extrinsic pressures [3,4]. TB is a common chronic deformation; in fact, TB may affect the quality of foot health because of the increase in physical fatigue, stress, inability to walk, pain, hyperkeratosis, and swelling of the external border of the distal lateral broadside of the fifth distal metatarsal [5].

TB occurs frequently in adolescents and adults, and more frequently in Caucasian versus African American people [6]. The prevalence of TB in the population is 13.8%, with a mean age of 45 years, and has a higher incidence of 69% to 79.5% in women [5,7,8,9]. Although, TB etiology is not clear because it appears to be multifactorial and is caused by an increase in friction and pressure on the lateral broadside of the foot from wearing tight shoes [5,10], a prominent lateral condyle which produces the hypertrophy of soft tissue [11], lateral bending of the fifth metatarsal [12], a short fifth metatarsal [13], accessory ossicles between the fourth and fifth metatarsals [7], and a biphalangeal fifth toe [14].

Alterations in metatarsophalangeal joints and toe deformities have been identified as a great public health disease with progressively escalating injury patterns [15]. TB is described as a condition with a strong relation to hallux valgus [16], and this relationship suggests remarkable foot disease, such as twisting, palpitating, or pain in the head of the fifth metatarsal [17,18], worse physical yield, and negative effects on daily life [19]. Nevertheless, the influence of the different degrees of TB types and their impact on health and quality of life, and the effects of oscillating degrees of TB on foot health-related quality of life are not well-clarified.

The objective of the research was to analyze and correlate the influence of the different types of TB on both foot health and general quality of life. Our hypothesis is that the degree of TB severity will decrease the quality of life of the persons who present this condition.

## 2. Materials and Methods

### 2.1. Design

A case–control research study was performed according to the Strengthening the Reporting of Observational Studies in Epidemiology (STROBE) criteria [20]. This study was approved by the ethics committee and biosecurity of Extremadura University (code: 124/2016, approved on 10 November 2016) and the Helsinki Declaration. All human experimentation rules were followed [21].

### 2.2. Participants

**Figure 1:**
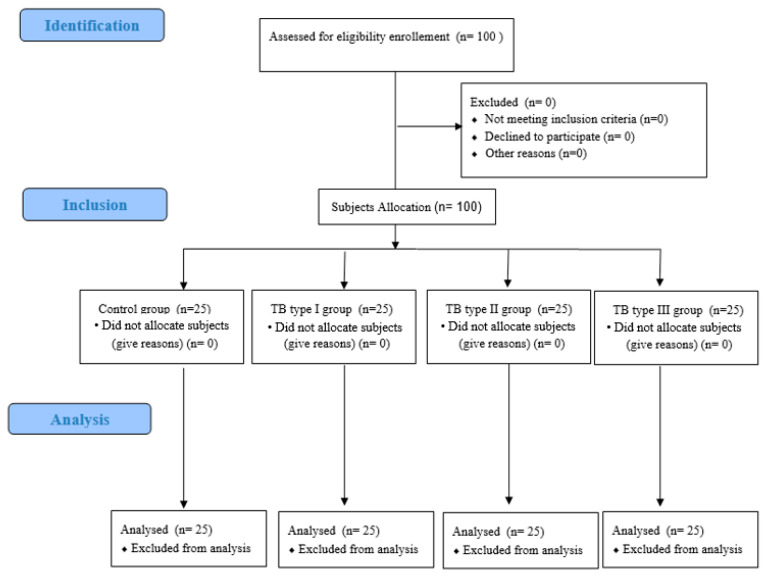
Study enrollment flow chart. Strengthening the Reporting of Observational Studies in Epidemiology (STROBE). TB = Tailor’s bunion.

The research sample consisted of 100 subjects. Data acquisition was performed between September 2019 and February 2020. The study was conducted in a podiatric surgery clinic at the University of Extremadura in the city of Plasencia (Spain) specializing in the treatment of foot diseases and disorders. To calculate a suitable size for the study sample, consecutive and non-random sampling was used to select 100 adult patients who attended the podiatry clinic with foot disorders that were matched to the characteristics of the study. All subjects who participated in the study signed an informed consent. Inclusion criteria consisted of several parameters: (1) adult participants with ages ranging from 21 to 92 years [22], and (2) in good health. Participants for the control group had no TB (n = 25), whereas study subjects presented with TB abnormalities, such as TB types I, II, and III (n = 25 in each group) [23] (Figure 1). The exclusion criteria included subjects with medical histories of immunosuppression, neurovascular disorders, neurological conditions, trauma or foot surgery, other foot deformities (plantar fasciopathy, plantar heel pain, metatarsalgia, hallux abductus valgus, toe deformities, plantar neuromas), not completing the writing tasks by oneself, and inability to comply with guidelines about the research and/or accomplish the study requirements [24,25].

### 2.3. Procedure and Measurements of FSHQ Results 

During the first visit, weight and height were measured by a qualified clinical analyst for all subjects. Subjects wore light, comfortable clothing and were barefoot. Quetelet’s equation was used to calculate body mass index (BMI) from the height (m) and weight (BMI = weight/height^2^) [26]. Next, participants completed the Foot Health Status Questionnaire (FHSQ) [27]. The FHSQ is used to evaluate different aspects related to foot pain, disability and restrictions in normal activities, quality of life, morphology, pain quantification, and both foot function and health [28]. The FHSQ consists of 19 items and is subdivided into five specific sections: (1) foot pain (four items), (2) foot function (two items), (3) foot health (three items), (4) footwear (three items), and finally, (5) overall health (general health, physical activity, social capacity, and vigor). The questionnaire was composed of 30 questions scored with a Likert scale that collects four foot health-related dimensions. The final score for each dimension is a numerical value between 0 and 100, with values closest to 0 representing worse quality of life. Each dimension analyzes certain functions: (1) The foot pain section contained questions about the type, severity, and duration of pain. A final score close to 0 indicated the presence of acute pain, while values close to 100 indicated the absence of pain. (2) The foot function section addressed the impact of foot health and its function in physical activities. Scores close to 0 indicated severe limitations in walking and working with general mobility restrictions. (3) The foot health section concerned personal perceptions about foot health. Scores close to 0 indicated a poor foot health condition, while scores close to 100 indicated an excellent foot condition. (4) The footwear section concerned adaptation to the type of footwear. Scores close to 0 indicated limitations in the type of footwear that could be worn, while scores close to 100 indicated that there were no restrictions on types of footwear. (5) The overall health section addressed the person’s general health condition. Scores close to 0 indicated poor overall health, while scores close to 100 indicated excellent health-related quality of life [28,29].

**Figure 2:**
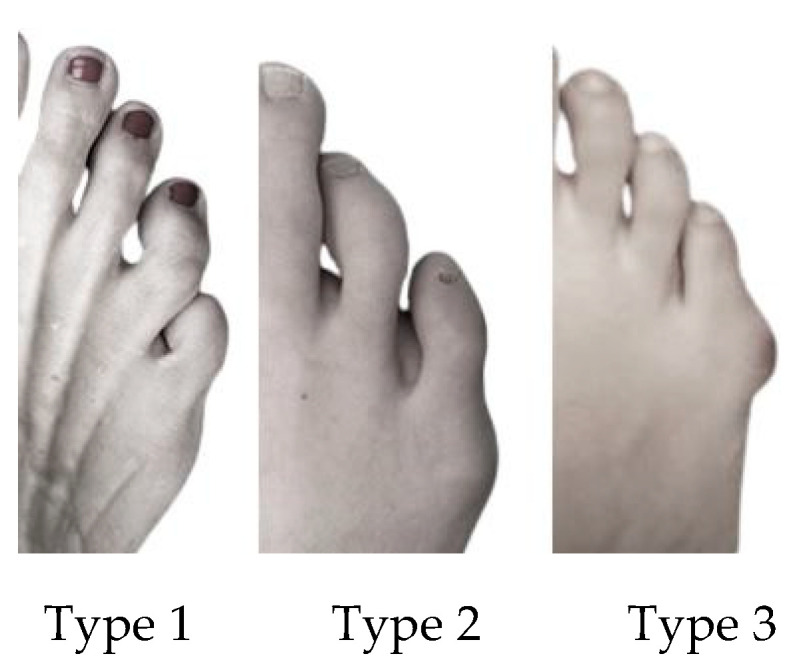
Coughlin Scale types. Type I: Enlarged 5th metatarsal head or lateral exostosis. Type II: Congenital bow of 5th metatarsal, normal 4th–5th intermetatarsal angles. Type III: Increased 4th and 5th intermetatarsal angle.

TB was evaluated using the Coughlin Scale [24,30] (Figure 2). This is used to determine three types of TB: (1) in type 1, the head of the 5th metatarsal is thickened and enlarged; (2) in type 2, a 5th metatarsal with an increased lateral curve and a normal fourth and fifth intermetatarsal angle (IMA) exists; and (3) type 3 has the greatest divergent position of the 5th metatarsal compared to the 4th metatarsal, and the 4th and 5th inter-metatarsal angles (IMAs) are increased. Generally, the most symptomatic TB is type 3.

### 2.4. Sample Size

To calculate the sample size by the one-way and TB deformity degree sample size, G*Power 3.1.9.2 software (Heinrich-Heine-Universität Düsseldorf, Düsseldorf, Germany) was used after considering a two-tailed hypothesis, a moderate effect size of 0.4, an alpha error of 0.05, and a power of 80% [31,32]. Consequently, a sample size consisting of at least 94 subjects was calculated. Ultimately, a total sample size of 100 subjects with 25 in each group was included in this study. 

### 2.5. Demographic and Social Descriptive Data

The demographic and descriptive data consisted of age, height, weight, BMI, and gender. Social data were composed of professional activity that was further subdivided into several categories: (1) student, (2) freelance, (3) employed worker, (4) unemployed, or (5) retired. Education level was subdivided into five categories: (1) incomplete primary, (2) complete primary, (3) secondary, (4) degree, or (5) superior degree. Civil status was subdivided into five categories: (1) single, (2) divorced, (3) widowed, (4) couple, or (5) married. Additional information concerning any underlying diseases or risk factors for foot disease (such as diabetes mellitus, use of systemic antibiotics or corticosteroids, immunosuppressants, vascular disorders, trauma, osteoarticular pathology), sports practice, and finally, TB and the side of foot disease (left, right, or both) were collected.

### 2.6. Statistical Analysis

Sociodemographic characteristics consisted of participants’ age, height, weight, and BMI. Independent variables were summarized as mean and standard deviation (SD), and maximum and minimum values were compared between varying types of TB. For the normality distribution of the variables, the Kolmogorov–Smirnov test was used, and data were considered as normally distributed if *p* > 0.05. Measurements for non-normally distributed data were analyzed with the nonparametric Kruskal–Wallis test to contrast differences among TB types. Finally, to contrast quantitative data by gender and FHSQ, an independent Student’s t-test was used to establish statistically significant differences. Mann–Whitney U tests were used for the nonparametric data. The Chi-squared test was used for categorical variables and to resolve differences among the observed frequencies. In order to eliminate systematic differences among the categories (foot function, foot pain, footwear, general foot health, general health, physical activity, social capacity, and vigor) of the FHSQ and different TB types, the Kruskal–Wallis test was used.

For all analyses, statistical significance was set at a *p*-value of <0.05 with a 95% confidence interval (CI). All analyses were performed with the statistical software SPSS version 21.0 (SPSS Inc., Chicago, IL, USA).

## 3. Results

### 3.1. Demographic, Descriptive, and Social Data

The research consisted of a total of 100 participants with ages ranging from 21 to 92-years-old, and the mean age was 51.70 ± 17.78. The sample by gender consisted of 39% females and 61% males. Table 1 presents demographic and descriptive results of the participants. Worth noting is that most patients were overweight (BMI = 26.02 ± 4.24 kg/m^2^) with a high prevalence in females (BMI = 27.36 ± 3.88 kg/m^2^). The statistical differences by gender (*p* < 0.05) included height and age rather than weight and BMI.

On the other hand, in Table 2, all the variables (civil status, study level, and professional activity) displayed statistically significant differences (*p* < 0.05).

### 3.2. Demographic, Descriptive, and Social Data by TB Classification 

With regard to the demographic and descriptive data based on TB classification, Table 3 indicates a high increase in BMI, weight, and age in TB3 participants. 

In Table 4, a high frequency of TB3 in the data occurred in married males (17, 68%), those with a civil status (13, 52%), incomplete primary study level (9; 36%), and those retired from professional activity (18, 72%).

### 3.3. FSHQ Male and Female Distribution and TB Classification

Table 5 displays the FHSQ foot disease data among females and males. Males with foot diseases displayed statistically significant differences (*p* < 0.05) for lower results in the sections of foot pain, foot function, footwear, foot and general health, and physical activity. The section concerning social capacity and vigor did not display any statistically significant differences (*p* > 0.05). 

Table 6 displayed the data comparison among the three types of TB according to the Coughlin scale and FHSQ scores. TB3 scores displayed the lowest values in foot pain, foot function, footwear, foot and general health, and physical activity. TB2 scores displayed the lowest values in social capacity and vigor.

### 3.4. Systematic Differences among FHSQ and TB Classification

Table 7 displays statistically significant differences (*p* < 0.05) in all groups for foot pain, foot function, footwear, foot health and general health, and physical activity, except in the control group (CG) vs. TB1 physical activity. Social capacity and vigor displayed non-statistically significant differences (*p* > 0.05).

## 4. Discussion

An increase in the types of TB is considered an important disturbance to quality of life and the impacts of foot deformity [33]. TB is a disorder associated with forefoot alteration with a large metatarsal area and a strong relationship with types of shoes worn that can produce gait disorders and cause an increase in the rate of falls [34,35]. During the first visit, we analyzed the influence of different TB types in relation to general quality of life and foot health in an adult sample. This finding is normally related to women [9,36]. These problems or conditions tend to increase among older people [9], and women have significant foot health conditions that require costly forefoot surgeries [37].

TB etiology is multifactorial and polemical. Even though there is no direct relationship between symptomatic TB and gender, this condition seems to occur more frequently in women [38]. The type and characteristics of footwear affect TB development, which occurs most frequently in women [9,36]. The results of our study demonstrate that men with TB3 showed the lowest values on the foot-related FSHQ score, proving that men have a greater limitation in contrast to that found in women.

TB3 is the most usual type in patients with symptomatic bunionettes, making the fourth and fifth intermetatarsal angles the determinants more likely to play an important role in people that showed symptomatic bunionettes [39].

Additionally, a decrease in plantar arch increases the risk of TB and hallux valgus [40]. Shi et al. demonstrated that subjects with flatfoot have a coexisting relationship between hallux valgus and TB, and hallux valgus shares common risk factors with TB deformity [4]. Deveci et al. demonstrated a relationship between hallux valgus and TB [16]. Recent studies in the literature have demonstrated that elderly people with different hallux valgus deformities showed a progressive decrease in foot and general health with an increase in the severity of hallux valgus that is independent of gender, and supported that modifying grades of hallux valgus affects social welfare in relation to foot health [23,41].

It appears difficult to collate the effects of these outcomes with other TB studies due to discrepancies in evaluation criteria. There is a specific lack of studies about TB and quality of life and foot health. Most of the research relates forefoot injuries, such as hallux valgus, hammer toes, TB, and metatarsal pain, with quality of life and foot health in general [9,42,43]. We have not found any scientific literature linking social well-being to foot health in patients with TB. For future research, we suggest more studies which focus only on the relationship between TB, foot health, and quality of life.

We found significant research limitations and restrictions which should be discussed. Our findings showed statistically significant differences for most FHSQ domains with a low sample size, even though our sample size calculation was carried out for a moderate effect size. The comparison between healthy controls and study cases, and the presence of sociodemographic differences among the groups, could have influenced these differences. Mainly, footwear types and characteristics that are related to morphological foot structure were limited with respect to family history; thus, this study analyzed these issues in addition to the increase in variability in the number of subjects, including participants from other countries. In this way, more credibility and a higher strength of research was achieved, which helped determine whether there was a specific sample for which the causes involved in deformity and general health could be analyzed. Advanced research should consider or allow for other elements, such as ethnicity, place, footwear, and/or social and economic conditions concerning the causes of TB related to a particular quality of life.

## 5. Conclusions

It was possible to carry out a verifiable measurement between the different types of TB with an alteration in quality of life related to foot health. The study results showed that factors such as age and footwear worn throughout life significantly influence the development of TB3 and its impact on foot pain and foot health.

## Figures and Tables

**Table 1:** Demographic and descriptive data of the sample population by sex.

Demographic and Descriptive Data	Total Group n = 100	Male n = 61	Female n = 39	*p*-Value
	Mean ± SD (Range)	Mean ± SD (Range)	Mean ± SD (Range)	
Age (years)	51.70 ± 17.78 (48.19–55.24)	51.78 ± 19.72 (47.53–58.01)	51.61 ± 19.72 (45.21–58.01)	<0.001 †
Weight (Kg)	82.55 ± 13.60 (79.85–85.25)	86.81 ± 10.76 (84.06–89.57)	81.53 ± 12.78 (77.38–85.67)	*0.189* †
Height (cm)	1.77 ± 0.80 (1.75–1.78)	1.83 ± 0.52 (1.81–1.84)	1.73 ± 0.77 (1.70–1.75)	*0.002* †
BMI (Kg/m^2^)	26.02 ± 4.24 (25.17–26.86)	25.92 ± 4.27 (24.06–27.76)	27.36 ± 3.88 (26.10–28.62)	*0.113* *

Abbreviations: BMI—body mass index; SD—standard deviation; range (min–max); * Mean; Student’s *t*-test for independent samples were applied; † Median ± interquartile range, *p* < 0.05 (with a 95% confidence interval) was considered statistically significant.

**Table 2:** Social characteristics of the sample by sex.

Social Characteristics		Total Group n = 100	Male n = 61	Female n = 39	*p*-Value
Civil Status	Single	23 (23%)	13 (21.3%)	10 (25.6%)	*0.009*
	Divorced	5 (5%)	2 (3.3%)	3 (7.7%)	
	Widowed	14 (14%)	6 (9.8%)	8 (20.5%)	
	Couple	6 (6%)	5 (8.2%)	1 (2.6%)	
	Married	52 (52%)	35 (57.4%)	17 (43.6%)	
Study Level	I. Primary	17 (17%)	11 (18%)	6 (15.40%)	*0.041*
	C. Primary	21 (21%)	14 (23%)	7 (17.90%)	
	Secondary	22 (22%)	9 (14.80%)	13 (33.30%)	
	Degree	14 (14%)	9 (14.80%)	5 (12.80%)	
	S. Degree	26 (26%)	18 (29.50%)	8 20.50%)	
Professional Activity	Student	5 (5%)	2 (3.3%)	3 (7.7%)	*0.031*
	Freelance	18 (18%)	10 (16.4%)	8 (20.5%)	
	Employed	35 (35%)	22 (36.1%)	13 (33.3%)	
	Unemployed	6 (6%)	5 (8.2%)	1 (2.6%)	
	Retired	36 (36%)	22 (3, 6.1%)	14 (35.9%)	

Abbreviations: C—complete; I—incomplete; S—superior. Chi-squared tests were utilized. In all the analyses, *p* < 0.05 (with a 95% confidence interval).

**Table 3:** Demographic and descriptive data of the sample population according to classification of tailor’s bunion.

Demographic and Descriptive Data	Total Group n = 100	CG n = 25	TB1 n = 25	TB2 n = 25	TB3 n = 25	*p*-Value
	Mean ± SD (Range)	Mean ± SD (Range)	Mean ± SD (Range)	Mean ± SD (Range)	Mean ± SD (Range)	
Age (years)	51.70 ± 17.78 (48.19–55.24)	44.28 ± 15.53 (37.86–50.69)	48.00 ± 15.07 (41.77–54.22)	49.80 ± 19.21 (41.86–57.73)	64.80 ± 14.58 (58.78–70.81)	*<0.001* †
Weight (Kg)	72.55 ± 13.60 (69.85–75.25)	74.12 ± 10.94 (69.60–78.63)	70.68 ± 14.67 (64.62–76.73)	70.06 ± 16.30 (63.33–76.79)	75.36 ± 13.60 (69.85–75.25)	*0.189* †
Height (cm)	1.67 ± 0.80 (1.65–1.68)	1.72 ± 0.88 (1.69–1.76)	1.64 ± 0.73 (1.61–1.67)	1.65 ± 0.62 (1.62–1.67)	1.65 ± 0.69 (1.62–1.68)	*0.002* †
BMI (Kg/m^2^)	26.02 ± 4.24 (25.17–26.86)	25.12 ± 3.05 (23.86–26.38)	25.89 ± 4.50 (24.03–27.75)	25.32 ± 4.45 (23.48–27.15)	27.74 ± 4.52 (25.87–29.61)	*0.113* *

Abbreviations: BMI, body mass index; TB: tailor bunion; CG: control group; SD: standard deviation; BMI: body mass index; range (min–max). One-way ANOVA was used † Median ± interquartile range, range (min–max) and * Mann–Whitney U test were used. *p* < 0.05 (with a 95% confidence interval) was considered statistically significant. Cougling Scale tool was used in tailor bunion’s classification.

**Table 4:** Demographic and descriptive data of the sample population according to classification of tailor’s bunion.

Social Characteristics		Total Group n = 100	CG n = 25	TB1 n = 25	TB2 n = 25	TB3 n = 25	*p*-Value
		Mean ± SD (Range)	Mean ± SD (Range)	Mean ± SD (Range)	Mean ± SD (Range)	Mean ± SD (Range)	
Sex	Male	61 (61%)	9 (36%)	17 (68%)	18 (72%)	17 (68%)	*0.031*
	Female	39 (39%)	16 (64%)	8 (32%)	7 (28%)	8 (32%)	
Civil Status	Single	23 (23%)	10 (40%)	5 (20%)	7 (28%)	1 (4%)	*0.009*
	Divorced	5 (5%)	2 (8%)	1 (4%)	2 (8%)	0 (0%)	
	Widowed	14 (14%)	2 (8%)	1 (4%)	2 (8%)	9 (36%)	
	Couple	6 (6%)	0 (0%)	1 (4%)	3 (12%)	2 (8%)	
	Married	52 (52%)	11 (44%)	17 (68%)	11 (44%)	13 (52%)	
Study Level	I. Primary	17 (17%)	1 (4%)	2 (8%)	5 (20%)	9 (36%)	*0.041*
	C. Primary	21 (21%)	3 (12%)	8 (32%)	4 (16%)	6 (24%)	
	Secondary	22 (22%)	10 (40%)	2 (8%)	6 (24%)	4 (16%)	
	Degree	14 (14%)	3 (12%)	5 (20%)	4 (16%)	2 (8%)	
	S. Degree	26 (26%)	8 (32%)	8 (32%)	6 (24%)	4 (16%)	
Professional activity	Student	5 (5%)	2 (8%)	1 (4%)	2 (8%)	0 (0%)	*0.031*
	Freelance	18 (18%)	6 (24%)	6 (24%)	4 (16%)	2 (8%)	
	Employed	35 (35%)	10 (40%)	11 (44%)	11 (44%)	3 (12%)	
	Unemployed	6 (6%)	2 (8%)	1 (4%)	1 (4%)	2 (8%)	
	Retired	36 (36%)	5 (20%)	6 (24%)	7 (28%)	18 (72%)	

Abbreviations: C—complete; I—incomplete; S—superior; TB—tailor’s bunion; CG—control group; ± SD—standard deviation (min–max). Kruskal–Wallis test was used. Frequency, percentage (%), and Chi-squared tests (χ^2^) were utilized. In all the analyses, *p* < 0.05 (with a 95% confidence interval) was considered statistically significant. Coughlin scale tool was used in the classification of tailor’s bunion.

**Table 5:** Comparisons of Foot Health Status Questionnaire scores by sex.

FSHQ Domains	Total Group n = 100	Male n = 61	Female n = 39	*p*-Value
	Mean ± SD (Range)	Mean ± SD (Range)	Mean ± SD (Range)	
Foot pain	63.46 ± 27.76 (57.95–68.97)	59.48 ± 26.67 (52.65–66.31)	69.69 ± 28.62 (60.41–78.97)	*0.042*
Foot function	77.06 ± 25.17 (72.06–82.05)	75.81 ± 26.38 (69.06–82.57)	79.00 ± 23.36 (71.43–86.57)	*0.517*
Footwear	39.25 ± 33.42 (32.61–45.88)	29.91 ± 29.04 (22.47–37.35)	53.84 ± 34.93 (42.52–65.16)	*0.001*
Foot health	46.37 ± 32.42 (39.94–52.80)	40.98 ± 31.67 (32.87–49.09)	54.80 ± 32.16 (44.38–65.23)	*0.032*
General health	72.00 ± 27.81 (66.48–77.51)	70.65 ± 28.85 (63.26–78.04)	74.10 ± 26.33 (65.57–82.64)	*0.590*
Physical activity	84.88 ± 20.25 (80.86–88.90)	82.33 ± 22.48 (76.57–88.09)	88.88 ± 15.60 (83.82–93.94)	*0.112*
Social capacity	78.87 ± 22.93 (74.32–83.42)	77.04 ± 24.06 (70.88–83.21)	81.73 ± 21.04 (74.90–88.55)	*0.391*
Vigor	60.43 ± 19.72 (56.52–64.35)	56.14 ± 20.04 (51.01–61.28)	67.14 ± 17.42 (61.49–72.79)	*0.018*

Abbreviations: FHSQ—Foot Health Status Questionnaire; SD—standard deviation. *p* < 0.05 with a 95% confidence interval was considered statistically significant. Mann–Whitney U tests were used.

**Table 6:** Comparisons of Foot Health Status Questionnaire scores between different classifications of tailor’s bunion.

FSHQ Domains	Total Group n = 100	CG n = 25	TB1 n = 25	TB2 n = 25	TB3 n = 25
	Mean ± SD (Range)	Mean ± SD (Range)	Mean ± SD (Range)	Mean ± SD (Range)	Mean ± SD (Range)
Foot pain	63.46 ± 27.76 (57.95–68.97)	89.30 ± 10.46 (84.98–93.61)	72.07 ± 21.61 (63.15–80.99)	54.10 ± 24.02 (44.18–64.01)	38.40 ± 22.53 (29.09–47.70)
Foot function	77.06 ± 25.17 (72.06–82.05)	94.75 ± 7.58 (91.61–97.88)	85.25 ± 17.75 (77.91–92.58)	67.75 ± 24.38 (57.68–77.81)	60.50 ± 29.57 (48.29–72.70)
Footwear	39.25 ± 33.42 (32.61–45.88)	67.66 ± 26.16 (56.86–78.46)	31.00 ± 34.06 (16.93–45.06)	31.66 ± 30.04 (19.26–44.06)	26.66 ± 26.89 (15.56–37.76)
Foot health	46.37 ± 32.42 (39.94–52.80)	76.90 ± 24.70 (66.70–87.09)	46.30 ± 29.85 (33.97–58.62)	38.70 ± 29.85 (26.37–51.02)	23.60 ± 19.27 (15.64–31.55)
General health	72.00 ± 27.81 (66.48–77.51)	84.40 ± 22.00 (75.31–93.48)	73.20 ± 23.75 (63.39–83.00)	68.00 ± 30.00 (55.61–80.38)	62.40 ± 31.12 (49.55–75.24)
Physical activity	84.88 ± 20.25 (80.86–88.90)	95.33 ± 10.10 (91.16–99.50)	89.33 ± 14.24 (83.45–95.21)	79.77 ± 26.19 (68.96–90.59)	75.11 ± 20.97 (66.45–83.77)
Social capacity	78.87 ± 22.93 (74.32–83.42)	86.00 ± 19.20 (78.07–93.92)	83.50 ± 22.16 (74.35–92.64)	72.50 ± 26.26 (61.65–83.34)	73.50 ± 21.74 (64.52–82.47)
Vigor	60.43 ± 19.72 (56.52–64.35)	69.25 ± 17.20 (62.14–76.35)	61.00 ± 16.36 (54.24–67.75)	54.00 ± 22.88 (44.55–63.44)	57.50 ± 19.59 (49.41–65.59)

Abbreviations: FHSQ—Foot Health Status Questionnaire; TB—tailor’s bunion; CG, control group; SD—standard deviation. Kruskal–Wallis test was used. Coughlin scale tool was used in the classification of tailor’s bunion.

**Table 7:** Systematic differences between Foot Health Status Questionnaire and patients with different classifications of tailor’s bunion.

FSHQ Domains	Post-Hoc p		
	CG vs. TB1	CG vs. TB2	CG vs. TB3
Foot pain	0.001	<0.001	<0.001
Foot function	0.023	<0.001	<0.001
Footwear	0.033	0.014	0.005
Foot health	0.002	<0.001	<0.001
General health	0.040	0.045	0.036
Physical activity	0.067	0.039	0.001
Social capacity	0.726	0.123	0.072
Vigor	0.125	0.054	0.096

Abbreviations: FHSQ—Foot Health Status Questionnaire; TB—tailor’s bunion; CG, control group; SD—standard deviation. *p* < 0.05 with a 95% confidence interval was considered statistically significant. Kruskal–Wallis test was used.

## Data Availability

The data that support the findings of this study are available from the corresponding author, upon reasonable request.
